# Cold Compress for Intrauterine Device Insertional Pain: A Randomized Control Trial

**DOI:** 10.1089/whr.2020.0056

**Published:** 2020-08-04

**Authors:** Jordan Hylton, Sarah Milton, Adam P. Sima, Nicole W. Karjane

**Affiliations:** ^1^ Department of Obstetrics and Gynecology, Virginia Commonwealth University Health Systems, Richmond, Virginia, USA.; ^2^ Department of Biostatistics, Virginia Commonwealth University, Richmond, Virginia, USA.

**Keywords:** contraception, insertional pain, intrauterine device

## Abstract

***Background:*** Pain with intrauterine device (IUD) insertion is identified as a barrier to uptake of this highly effective long-acting reversible contraceptive. Several studies have assessed the efficacy of interventions to alleviate patient discomfort associated with IUD insertion, but no interventions have been clearly shown to improve procedural pain. The aim of this study was to determine whether use of a cold compress on the abdomen during IUD insertion reduces pain.

***Materials and Methods:*** This was a prospective randomized control trial of women presenting to Virginia Commonwealth University for insertion of IUD from September 2016 to October 2017. A power analysis determined that 69 subjects were needed in each arm to detect a 30% reduction in pain with a power of 80%, significance value of *p* < 0.05. One hundred forty-two participants were consented for the study, 69 were randomized to the control group, which received the usual management, and 73 were randomized to the study group, which received a cold compress to the abdomen before the procedure. In addition to data on the difference from pre- to postprocedure pain scales, we collected information regarding inserting provider type, gravidity/parity, body mass index, demographic information (age, race, insurance type, and level of education), history of IUD placement or cervical procedure, history of chronic pain, and the use of regular pain medications (defined as more than once per week). Statistical analysis was accomplished using *t*-test and chi square tests.

***Results:*** There was no difference in pre and postinsertional pain in those who received a cold compress versus the control during insertion of an IUD (3.4 vs. 3.5). The insertional pain was rated at 4.3 and 4.6 for patients who received the cold compress and the control group, respectively (*p* = 0.805).

***Conclusion:*** Although a cold compress is a simple, inexpensive, and safe method of pain control, this study shows no reduction in insertional pain for IUD placement.

## Introduction

Intrauterine devices (IUDs) are a popular top-tier choice for women seeking contraception.^1,2^ In fact, they are one of the most widely used contraceptives in the world and are considered a first-line contraceptive option for women.^1–3^ Furthermore, The American Academy of Pediatrics and the American College of Obstetricians and Gynecologists now recommend long-acting reversible contraceptive devices (LARCs), like IUDs, as first-line therapies for sexually active adolescents.^1,4^ Despite this emphasis, unintended pregnancies account for ∼50% of all pregnancies in the United States, with ∼40% ending in induced abortion.^1,2^ Many studies have looked at barriers to contraception.^5–12^ In particular, pain with IUD insertion has been identified as a barrier to uptake of this highly effective LARC.^5^

There are several published studies assessing the efficacy of interventions to alleviate patient discomfort associated with IUD insertion. Nonsteroidal anti-inflammatory drugs reduced postinsertion pain but did not have an effect on peri-insertional pain.^11^ Misoprostol administration preinsertion facilitated placement but did not have an impact on pain.^10^ Paracervical block did not reduce peri- or postprocedure pain.^9^ A double-blinded study comparing topical viscous gel with topical lidocaine did not show reduction in pain with lidocaine administration.^7^ Most recently, a sham-controlled randomized trial compared paracervical block with control. This study showed an reduction of pain associated with the procedure, specifically with the pain associated with the tenaculum placement.^12^ These results continue to highlight the need for further research on effective analgesia during IUD insertion.

Ice or cold compresses have been used as an intervention to alleviate pain in a multitude of specialties. The pathophysiology by which cold compresses reduce pain is unclear. One plausible mechanism is antinociceptive effect of cold compresses on the gate control system. Although it is uncertain how local or widespread this affect may be, the propagation of this effect may decrease muscle spasms and nerve conduction.^8^ Several studies have shown cold compress to decrease periprocedural pain. In the sports medicine community, it is used widely and is considered a harmless and effective treatment for pain associated with disease and injury.^13^ In addition, a study designed to evaluate cold compress in the postoperative management of total knee arthroplasty concluded a dramatic reduction in blood loss and decreased necessity for narcotic analgesia.^14^ Most recently, in the *Journal of American Science*, cold compress was also noted to decrease the pain and anxiety level associated with removal of chest tubes.^15^

To our knowledge, there has not been a rigorous investigation into the use of a cold compress on pain during the insertion of an IUD. Our hypothesis was that the application of a cold compress to the abdomen during insertion of an IUD would decrease the pain associated with the procedure.

## Materials and Methods

We conducted a prospective nonblinded randomized controlled trial conducted at two clinics in Richmond, Virginia, from September 2016 until October 2017. The trial was registered with ClinicalTrials.gov (NCT02898831) and approved by the Virginia Commonwealth University institutional review board (IRB HM20004308).

Before the study, the biostatistician provided the study coordinator with a randomization schedule using a random number generator based on the uniform distribution. The randomization was carried out by research staff before the initiation of the study. We also performed a power analysis to determine sample size. Comparable studies that assess procedural pain considered a 30% reduction in pain to be significant. Based on these studies, we considered a 30% reduction in pain between the experimental and control groups to be significant.^7^ Assuming that the clinical difference in pain was 30%, this study required 69 patients in each group to achieve 80% power at the 0.05 level

**FIG. 1.**
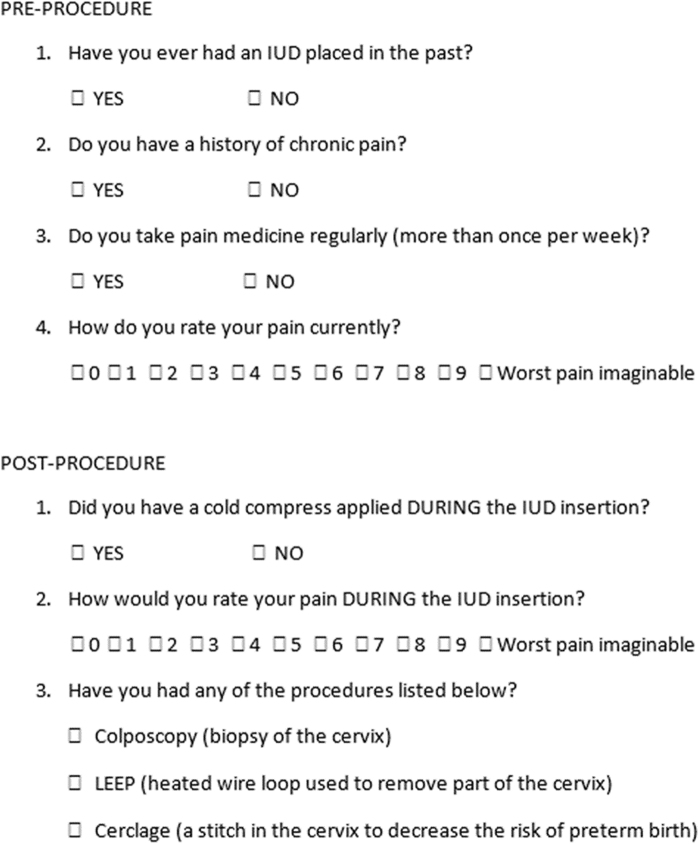
Questionnaire women were asked to complete both before and after the IUD insertion. IUD, intrauterine device.

Eligible participants included all nonpregnant women, 18 years of age or older, presenting to the VCU Women's Health clinics for placement of any type of IUD. Subjects were excluded if they were decision impaired, imprisoned, non-English speaking, or under the age of 18 years. Potential subjects were identified at the time of presentation to the Women's Health Clinics at Virginia Commonwealth University Health Systems for IUD insertion. Those who agreed to participate and provided written informed consent were randomized to each of the two treatment arms by pulling sequentially numbered opaque envelopes containing the computer-randomized allocations. After randomization, the provider collected information regarding basic demographic data and comorbidities that may affect the patient's perception of pain from the subjects' electronic medical chart and completed the preprocedure questionnaire with the patient (Fig. 1) as well as a preprocedure 10-point visual acuity scale.

Subjects randomized to the cold compression group received a cold compress placed on the abdomen for 5 minutes before and throughout the IUD placement. Subjects randomized to the control group received routine care during the procedure. Neither group had a paracervical block performed during any point of the procedure. After IUD insertion, the patient completed a validated 10-point visual acuity scale for the evaluation of pain during the procedure, and the provider completed the remainder of the postprocedure questionnaire with the patient.

Two primary investigators collected the data, performed data entry, and reviewed charts to ensure accuracy of information provided by the inserting physician. They also performed periodic review of the data entry to ensure completeness and accuracy. This information was stored in the Virginia Commonwealth University REDCap database.

Subject characteristics were summarized using means and standard deviations (SDs) or frequencies and percentages. Separate summaries were provided for each treatment group. A two-sample *t*-test was used to compare the mean difference in self-reported pain scores pre and postinsertion. All inference was made at the 0.05 level. Statistical analysis was supported, in part, by award no. UL1TR002649 from the National Institutes of Health's National Center for Advancing Translational Science.

## Results

One hundred forty-one subjects consented to participate and enrolled in the study. Seventy four (52%) were randomized to the study group and 67 (48%) to the control group. Of these, two subjects randomized to the cold compress group and a single subject in the control group were younger than 18 years of age. In addition, one subject in the control group had an incomplete consent form. In each of these cases, the local IRB was contacted and these subjects were excluded from all analyses.

Subject demographic information is displayed in Table 1. Most participants were white (68%) and had private insurance (74%). The mean age was 30.9 years (SD = 7.7), with subjects ranging from age 18 to 51 years. Almost all (89%, *n* = 126) participants underwent placement of the Mirena IUD. Other IUD types included Skyla (*n* = 9, 6.4%) and Paragard (*n* = 6, 4.3%). Sixteen participants reported a history of chronic pain and 9% of all subjects were regularly taking pain medications. There were no clinically important differences between the treatment groups.

**Table 1.** Summary Characteristics

Characteristic	Level	Summary (N* = 138), *n (%)	Cold compress	Control
			Summary (N* = 66), *n (%)	Summary (N* = 72), *n (%)
Race	White	93 (68)	45 (63)	48 (74)
	Black	36 (26)	20 (27)	16 (25)
	Other	8 (6)	7 (10)	1 (2)
Age		30.9 (7.7)	30.4 (6.7)	31.4 (8.6)
Insurance	Private	102 (74)	48 (67)	54 (83)
	Medicaid	28 (20)	20 (28)	8 (12)
	Other	7 (5)	4 (6)	3 (5)
Education	HS or less	28 (31)	15 (33)	13 (29)
	Undergraduate	40 (44)	19 (41)	21 (47)
	Postgraduate	23 (25)	12 (26)	11 (24)
Provider	Attending Physician	101 (74)	52 (73)	49 (74)
	Resident or NP	36 (26)	19 (27)	17 (26)
Gravida	0	45 (33)	25 (35)	20 (31)
	1	31 (23)	15 (21)	16 (25)
	2	36 (26)	20 (28)	16 (25)
	>3	24 (18)	12 (17)	12 (19)
Para	0	46 (34)	25 (35)	21 (33)
	1	42 (31)	22 (31)	20 (31)
	2	37 (27)	18 (25)	19 (30)
	>3	11 (8)	7 (10)	4 (6)
IUD type	Mirena	123 (89)	64 (89)	59 (89)
	Other	15 (11)	8 (11)	7 (11)
Prior IUD	Yes	98 (71)	50 (69)	48 (73)
	No	40 (29)	22 (31)	18 (27)
Pain medication	Yes	125 (91)	66 (92)	59 (89)
	No	13 (9)	6 (8)	7 (11)

Frequencies may not sum to the total sample size due to missing data.

IUD, intrauterine device.

Before IUD insertion, the cold compress and control groups had mean pain scores of 0.8 (SD = 1.9) and 1.2 (SD = 2.0), respectively. After insertion, the pain scores increased to a mean of 4.3 (SD = 2.6) and 4.6 (SD = 2.5), respectively, in each of the cold compress and control groups. This resulted in a mean change scores of 3.4 (SD = 2.7) and 3.5 (SD = 2.5) in the two treatment arms, with a difference in the change scores of 0.1 (standard error = 0.4). The changes in the pain scores were not different between the treatment arms (*p* = 0.805).

Six participants in the cold compress group and nine in the control group indicated they had chronic pain. Overall, pain scores for participants indicating chronic pain were higher than those with no indication of chronic pain. Participants with chronic pain had mean pain levels of 2.5 (SD = 3.0) and 5.8 (SD = 2.7) at before and during insertion, respectively, compared with mean pain scores of 0.9 (SD = 1.7) and 4.3 (SD = 2.5) for those without chronic pain. However, the change before and during insertion did not differ based on chronic pain (difference = 0.2, SD = 2.5; *p* = 0.882).

## Discussion

Strengths of this study include the randomized controlled study design and appropriate sample size. The study was adequately powered to detect a difference between the treatment groups, and no subjects were lost to follow-up. We also use a validated pain assessment tool and took into account potential confounding factors such as pain syndromes and prior cervical procedures. In addition, we focused difference in change of pain score noted before and after the procedure to account for the variations in perception of pain among participants.

Limitations of our study include the lack of documented tenaculum placement as well as confirmation of the timing of cold compress application. This would be of particular interest given prior discussions with regard to the improvement with alternative interventions such as a paracervical block. We also did not stratify for body mass index, which may have affected the impact of the cold compress on insertional pain. Furthermore, subjects were also not stratified for parity or delivery type. Alternative studies were stratified based on parity and delivery types and have found increased pain scores in women with prior cesarean sections when compared with those in prior vaginal birth and further in the nulliparous patient.^16,17^

As in many prior studies, it is difficult to assess pain in a single inciting event and we recognize that there are multiple components to a patients perception during a procedure. Another key limitation of our study design is that the research staff and study participants were not blinded to the group assignment. And, although chronic pain was considered as potential for differences in pain perception, we did not document the use of pain medications within the 24 hours before IUD insertion. We understand that the history of IUD use may also effect the anticipatory perception of pain at time of insertion, which we did not account for in our study. In addition, we did not separately assess for the use of smaller diameter devices, marketed specifically for nulliparous women. Although these devices were included in the study, there were too few to perform any meaningful analysis.

This study was performed to assess the impact of a low-cost low-risk intervention on the pain associated with the insertion of an IUD. Although this study approaches the call for nonpharmacologic options, it is increasingly important to address the impact that anticipatory anxiety can have perception of pain.^18^ In summary, cold compress placed on the abdomen before and throughout IUD insertion did not reduce pain associated with the procedure. Despite the theoretical benefit, this study demonstrated no difference in postprocedural pain and no difference in change of pain score noted before and after the procedure. Further research is warranted with consideration of perceptions of pain and in those with pain before the procedure. In addition, future studies should assess the role of both tenaculum placement and diameter of device placed in procedure-related pain as well as approaches to transpose anxiety related to the IUD insertion.^18^

## Abbreviations Used

IUD: intrauterine device
LARC: long-acting reversible contraceptive device
SD: standard deviation
